# TTADDA-UAV: A multi-season RGB and multispectral UAV dataset of potato fields collected in Japan and the Netherlands

**DOI:** 10.1016/j.dib.2025.112004

**Published:** 2025-08-23

**Authors:** Bart. M. van Marrewijk, Stephen Njehia Njane, Shogo Tsuda, Marcel van Culemborg, Gerrit Polder, Kenji Katayama, Tim van Daalen, Rick van de Zedde

**Affiliations:** a Wageningen University and Research, Wageningen, the Netherlands; b Research Centre for Agricultural Information Technology, National Agriculture and Food Research Organization (NARO), Japan; c Hokkaido Agricultural Research Centre, National Agriculture and Food Research Organization (NARO), Japan; d Solynta, Wageningen, the Netherlands

**Keywords:** Phenotyping, Yield estimation, Agriculture, Orthomosaic

## Abstract

The *Transition to a Data-Driven Agriculture (TTADDA)* project focuses on advancing the shift toward high-tech, circular agriculture. By developing cutting-edge sensor technologies and AI-driven tools, the project aims to boost productivity through a data-centric potato production system that supports circular agricultural practices. Potato phenotyping is crucial for creating high-yielding, resilient, and sustainable potato crops, which are essential in global food systems. Specifically in the Netherlands, the global leader in seed potato production and Japan that produces certified seed potatoes under strict quality controls and phytosanitary regulations. A multi-season drone dataset from five potato trials—three in Japan and two in the Netherlands was collected. Each trial field was divided into small plots, each planted with a specific cultivar to assess varietal performance. Data included drone imagery (RGB and multispectral), manual yield and ground coverage measurements, and weather data. The combination of sensor versatility, diverse potato varieties, and varying climate and soil conditions between Japan and the Netherlands makes this dataset highly valuable and potentially reusable for a wide range of applications. Using MIAPPE for this dataset ensures consistent, clear documentation of sensors, varieties, and conditions, making the data findable, reusable, and easy to integrate with other studies. It also supports reproducibility and automated analysis across the multi-location trials.

Specifications Table

**Table:** 

Subject	Biology
Specific subject area	*Briefly describe the specific subject area.* Max *150 characters (without spaces).* This dataset aims to boost research in potato phenotyping by publishing an international multi-season dataset.
Type of data	RGB orthomosaic (*rgb.tiff) Elevation orthomosaic (*dsm.tiff) Multi spectral orthomosaic (*msp.tiff or seperated for each band *blue.tiff) Raw -> ground truth data / measurements (.csv) - Yield - Ground coverage - Weather
Data collection	In this study, a multi-season drone dataset was collected from three potato trials in Japan and two in the Netherlands. Every trial consisted of a potato field, which was divided into smaller plots (±1.5 × 3 m). The plots were planted with a specific cultivar to evaluate and compare the performance and characteristics of different potato varieties. Data collection included drone imagery, manual measurements, and on-site weather data. Weekly RGB and multispectral images were processed with Agisoft Metashape to create a DEM, RGB and multispectral orthomosaics. Manual measurements covered yield and ground coverage. All data was summarized in the MIAPPE format to make the data FAIR.
Data source location	The TTADDA-UAV is a multi-season internation drone dataset. Therefore, geographical coordinates of each season was different: TTADDA_NARO_2021: latitude: 42.8818186, longitude: 143.0756943 TTADDA_NARO_2022: latitude: 42.888711, longitude: 143.0736002 TTADDA_NARO_2023: latitude: 42.888865279, longitude: 143.072670842 TTADDA_WUR_2022: latitude: 51.99146594, longitude: 5.580833384 TTADDA_WUR_2023: latitude: 51.9922293, longitude: 5.582514 Data is stored at data servers of the Dutch 4TU.Federation foundation (4TU.ResearchData).
Data accessibility	Repository name: The dataset is part of the following collection: Data identification number: doi.org.10.4121/936b5772–09fc–4856–983d-1f9cc2f38d15 Direct URL to data: (https://doi.org/10.4121/936b5772-09fc-4856-983d-1f9cc2f38d15) The collection consist of metadata, and five related studies: TTADDA_NARO_2021, TTADDA_NARO_2022, TTADDA_NARO_2023, TTADDA_WUR_2022, TTADDA_WUR_2023 To visualise the metadata and download the dataset we recommend the following GIT: https://github.com/NPEC-NL/MIAPPE_TTADDA_dataset
Related research article	None

## 1 Value of the Data

• This study was conducted within the public-private partnership TTADDA — Transition Towards a Data Driven Agriculture — a collaboration between Dutch and Japanese companies and research institutes. The project focuses on advancing high-tech, circular agriculture by using novel sensors and AI to boost productivity. Both countries face challenges like labour shortages, sustainability demands, and climate change, requiring solutions to increase yield through selecting more resilient potato varieties. Collaboration on tools, knowledge, and data is essential to support breeders in variety selection. The result is the TTADDA-UAV dataset, a unique multi-season resource for breeding and developing generalized crop models.
• An important contribution of the dataset is combination of different RGB and multispectral sensors, diverse potato varieties, manual measurements, varying climate and soil conditions between Japan and the Netherlands and most important the fact that imaging is done over different seasons including yield measurements, makes this dataset highly valuable. Three potential studies that could be conducted on presented dataset: 1) Improving crop models by relying on ground truth yield measurements of five different studies. 2) Development and validation of advanced UAV phenotyping pipelines by for example comparing estimated ground coverage with ground truth measurements. 3) Development of algorithms to assist breeders in selecting the most promising crops, levering the multi-seasonality of the TTADDA-UAV dataset.
• The TTADDA-UAV dataset makes it possible to study the effect of seasonality on varieties. Most European varieties favor late maturing, because of the relative long season in Europe, whereas in Japan, especially in the Hokkaido region the season is much shorter due the cold winters. In the TTADDA-UAV dataset multiple varieties are tested, enabling new insights in the influence of climate, rainfall and soil properties on yield.
• Standardisation of data is crucial to fully explore and derive traits of high-throughput phenotyping experiments. One of the available standards is the MIAPPE format, also known as Minimum Information About Plant Phenotyping Experiments. MIAPPE aims to standardize all variation of plant phenotyping experiment into a template to make data findable and reusable. [8]. Fairifying data is time consuming and difficult work [8]. Yet we believe metadata as added to this dataset is vital to make a dataset machine readable and reusable. To our best knowledge the number examples in UAV datasets in MIAPPE format is limited [6,10]. By publishing our dataset in the MIAPPE format, it can serve as a guideline for future UAV experiments. Additionally, using the MIAPPE standard ensures that all dataset components such as study descriptions, observation units, biological materials, and sensor information are accessible through the MIAPPE template. This makes the dataset FAIR (Findable, Accessible, Interoperable, and Reusable) and promotes its reuse in future research.

## 2 Background

Monitoring potato growth is essential for precise phenotyping, yield estimation, and disease management. Due to the crop’s complex canopy and ridge-based cultivation, traditional manual methods—such as SPAD meters for chlorophyll absorption [13] and handheld spectrophotometers for canopy growth [5]—are limited in scope and efficiency. These approaches risk damaging crops and cannot adequately capture field-wide variability.

Unmanned Aerial Vehicles (UAVs) offer an efficient alternative, allowing large-scale, cost-effective monitoring. UAVs have been used for estimating potato crop emergence [4], canopy coverage [2], and height for spray volume calculations [12]. Multispectral sensors enhance this capability by producing indices such as NDVI to assess crop greenness [1] and volume [7] or disease detection diseases [3,11].

The TTADDA-UAV dataset provides orthomosaics, DSM and manual measurements to validate trait extraction algorithms, like crop emergence and coverage. By improving these algorithms breeders can be assisted in variety selection based on quantitative data.. Moreover, the dataset contains location, variety and year specific yield measurements, enabling researchers to improve, calibrate and validate crop models on multi-seasonal dataset.

## 3 Data Description

### 3.1 MIAPPE format

An important contribution of this data paper is to publish the dataset in the Minimum Information About Plant Phenotyping Experiments (MIAPPE) format [8]. The MIAPPE consist of a hierarchical structure. In our dataset the MIAPPE excel sheet is included (“MIAPPE_TESTTEST.xlsx”. It contains seven structured sheets in line with the MIAPPE format:

- **Investigation**: In the investigation the main components of the dataset are described, including short description of the dataset, MIAPPE version, contact person, submission date.
- **Study**: The study sheet summarises all experiments. The TTADDA dataset is subdivided into 5 studies: TTADDA_NARO_2021, TTADDA_NARO_2022, TTADDA_NARO_2023, TTADDA_WUR_2022 and TTADDA_WUR_2023 each study representing a different season and location.
- **Observation Unit:** In this sheet fields and plot are described. Some observations like weather and drone data are measured on field level. Other observations, like the plant density, are recorded on plot level. Each observation unit is linked to a studyId.
- **Biological Material:** Describes the biological material, for example, *biologicalMaterialId*: Irish Cobbler, *organism: 4113, genus*: Solanum, *species*: Solanum tuberosum.
- **Data file:** This sheet contains links to all the images and data files. Each file has a specified *dataId* and location *dataFileLink* that contains observations. A file can either be an orthomosaic or for example a CSV file. A CSV file can store data for multiple observation units and several observed variables. For example, it may contain multiple rows identified by an observation unit ID (obsUnitId) and multiple columns representing variables such as tuber weight at harvest (tubwght_total_kgm-2) or dry matter content.
- **Sensor:** This sheet summarises all sensors used in the investigations, it is used to list which measurement was taken with which sensor.
- **Observed variables:** All observations on plot and field level are described/referenced in the observed variables sheet. In the TTADDA study many different observations are made:
  ○ Weather data, which has multiple observed variables: air_temp_avg, air_temp_max, air_rh_avg etc.
  ○ UAV data: RGB, DSM and multispectral orthomosaics
  ○ Measurements during the experiment: estimated ground coverage, day of yellowing, manual measured plot height and width
  ○ Destructive measurements: yield, dry matter content, starch content, fresh weight leaves, fresh weight stem etc.
- **Events:** Events are discrete occurrence at a particular time during the study. For example applying fertilizers or irrigation is only done once. Events are linked to a studyId and observation unit ID

### 3.2 Dataset structure

**Fig. 1:**
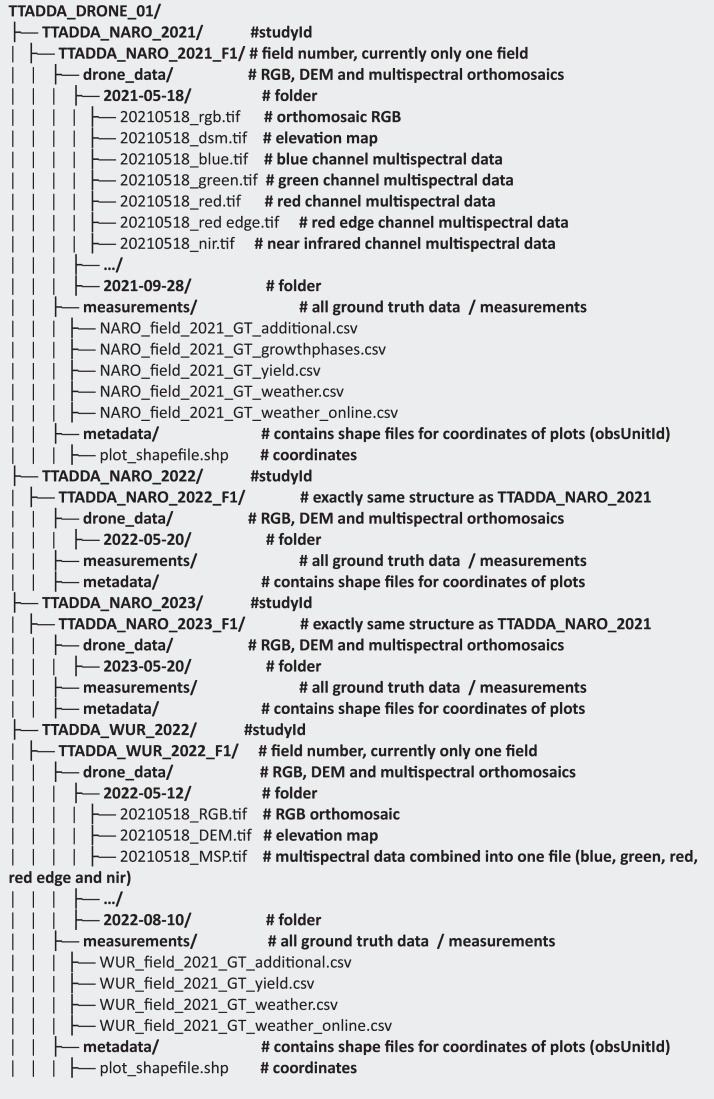
Summary of dataset structure. All files can be access from MIAPPE Excel file.

The data, approximately **550Gb** in size, can be downloaded from [link]. Fig. 1 provides an overview of the dataset structure, which follows the MIAPPE excel format. The dataset is organised into five main folders, each corresponding to a specific study: TTADDA_NARO_2021, TTADDA_NARO_2022, TTADDA_NARO_2023, TTADDA_WUR_2022 and TTADDA_WUR_2023, within each study folder, subfolder are organised by field number (Fx), and include drone_data, field measurements, and metadata.

### 3.3 Loading using MIAPPE

**Fig. 2:**
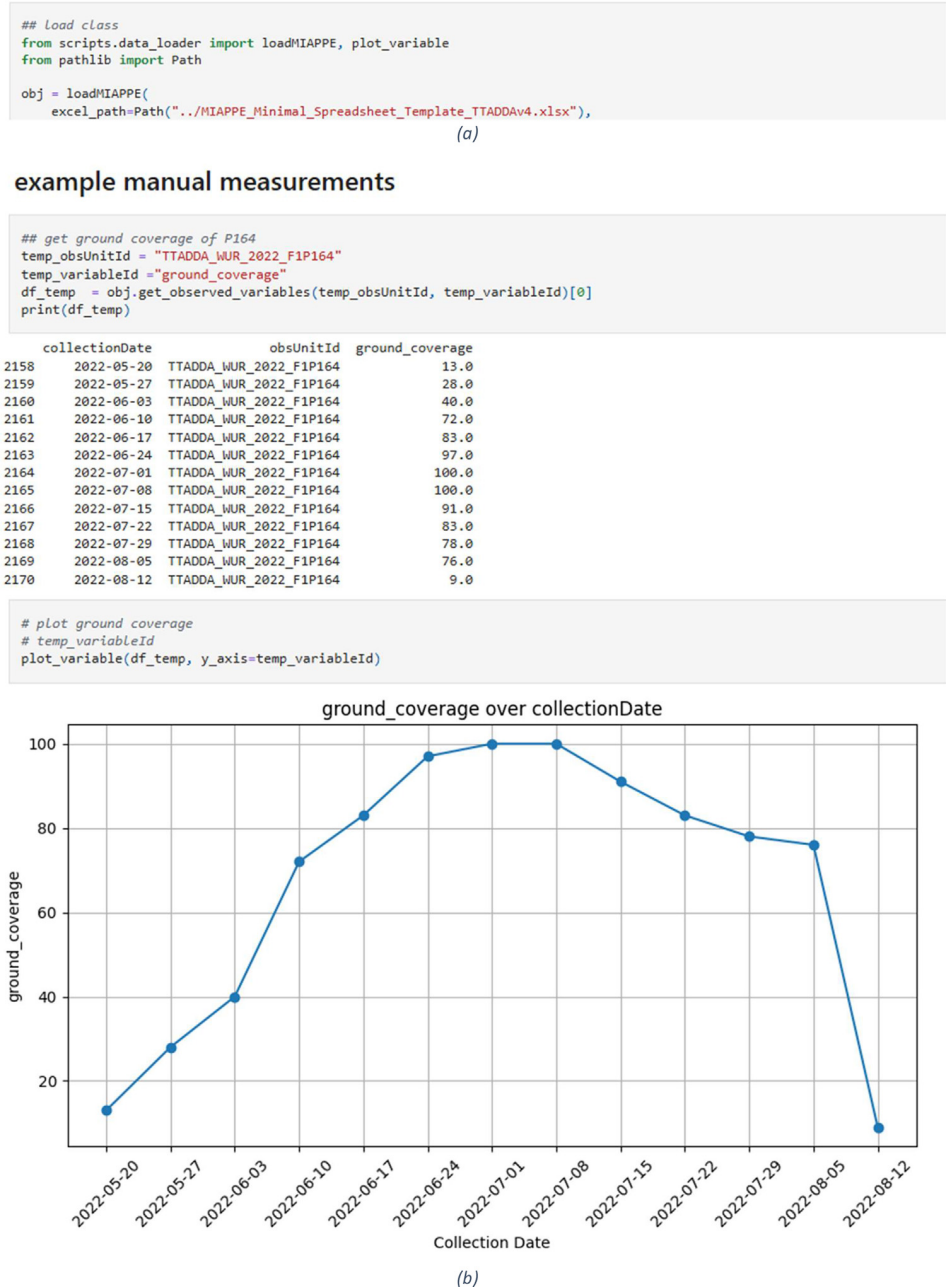
(a) example of loading MIAPPE excel sheet in python. (b) Example to get ground coverage data of specific observation unit.

For enabling fair data following git is recommended: https://github.com/NPEC-NL/MIAPPE_TTADDA_dataset. The GIT is meant to correctly load and visualize all data by using the MIAPPE format. An example is shown in Fig. 2.

## 4 Experimental Design, Materials and Methods

In this section, the five studies are explained in more detail.

### 4.1 TTADDA_NARO_2021

**Fig. 3:**
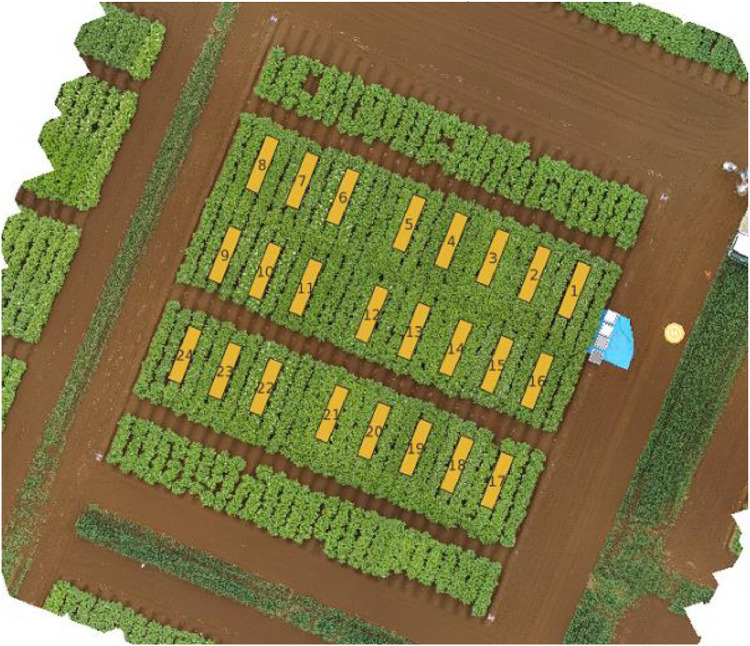
Field corresponding with studyId: TTADDA_NARO_2021.

The TTADDA_NARO_2021 study was conducted in the Memuro region of Japan (latitude: 42.8818186, longitude: 143.0756943). Most important properties are summarised in Table 1. Planting took place on May 18, 2021, and the harvest was completed on October 11, 2021. The field consisted of 24 plots (8 varieties), each measuring 0.75 m by 3 m and a plant density of 4.44 plants/m2. An overview of the field layout is shown in Fig. 3. The potatoes were planted by hand firstly cutting them into two and placing them with the sprout facing upwards at a spacing of 30 cm each and lightly covering with the soil. Finally, the rows were covered to form a ridge using a tractor-driven hiller thus forming ridges of 0.3 m height and 0.75 m interval between the ridges.

### 4.1 TTADDA_NARO_2022

**Fig. 4:**
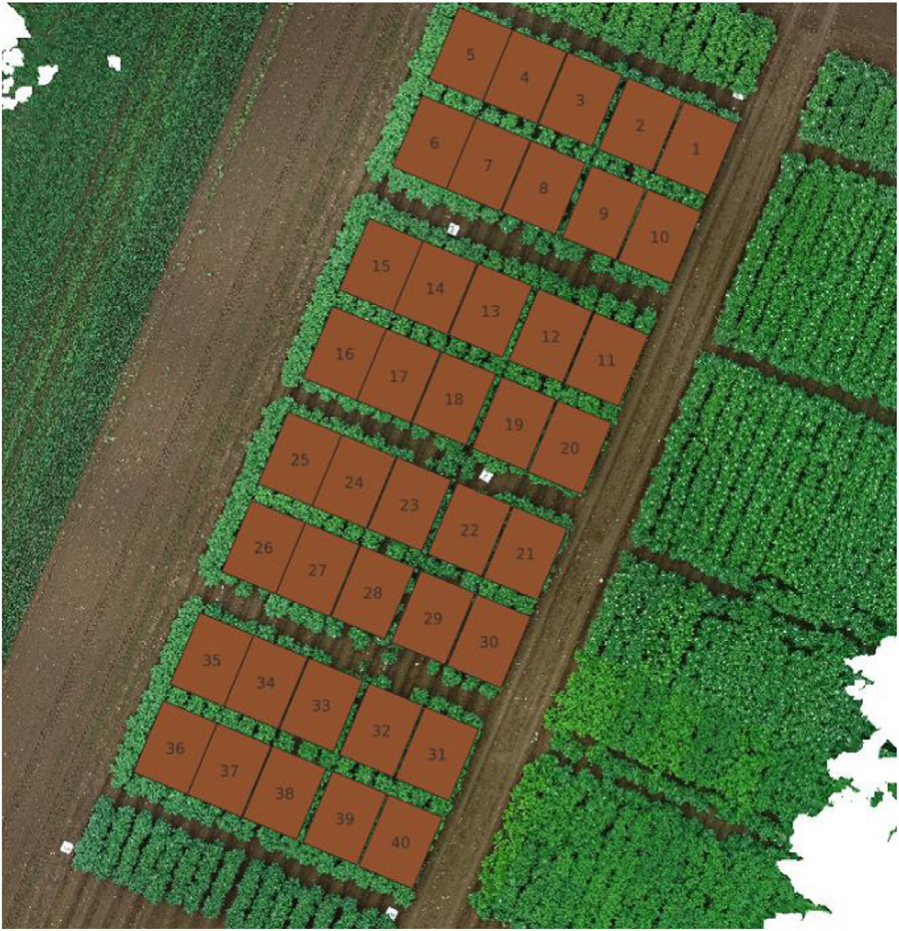
Field corresponding with studyId: TTADDA_NARO_2022.

The TTADDA_NARO_2022 study was conducted in the Memuro region of Japan (latitude: 42.888711, longitude: 143.0736002). Most important properties are summarised in Table 1 Table 2. Planting took place on May 20, 2022, and the harvest was completed on October 3, 2022. The field consisted of 40 plots (9 varieties), each measuring 2.25 m by 3 m and a plant density of 4.44 plants/m^2^. Similarly, as the previous year, the potatoes were hand planted, placed in a 0.30 m interval with the sprout facing upwards and lightly covered with the soil, after which ridge was formed by covering the potatoes in a 0.3 m high ridge with a 0.75 m interval. An overview of the field layout is shown in Fig. 4.

**Table 1:** Metadata of TTADDA_NARO_2021 field. *Values derived from SoilGrids (Poggio et al., 2021).

Field properties	
studyId	TTADDA_NARO_2021
Number of plots	24
Number of varieties	8
Plant density [plants/m2]	4.44
Ground sampling distance [mm/pixel]	7.8
Number of drone flights	19
**Soil properties**	
Clay [%]	11.0*
Sand [%]	56.8*
Silt [%]	32.3*
Ph [-]	5.9
Bulk density [kg/dm3]	0.74
Cation Exchange Capacity (CEC) [mmol+/kg]	172

**Table 2:** Metadata of TTADDA_NARO_2022 field. *Values derived from SoilGrids [9].

Field properties	
studyId	TTADDA_NARO_2022
Number of plots	40
Number of varieties	9
Plant density [plants/m2]	4.44
Ground sampling distance [mm/pixel]	4.0
Number of drone flights	19
**Soil properties** [9]	
Clay [ %]	10.8*
Sand [ %]	55.6*
Silt [ %]	33.7*
pH [-]	5.7
Bulk density [kg/dm3]	0.79
Cation Exchange Capacity (CEC) [mmol+/kg]	173

### 4.2 TTADDA_NARO_2023

**Fig. 5:**
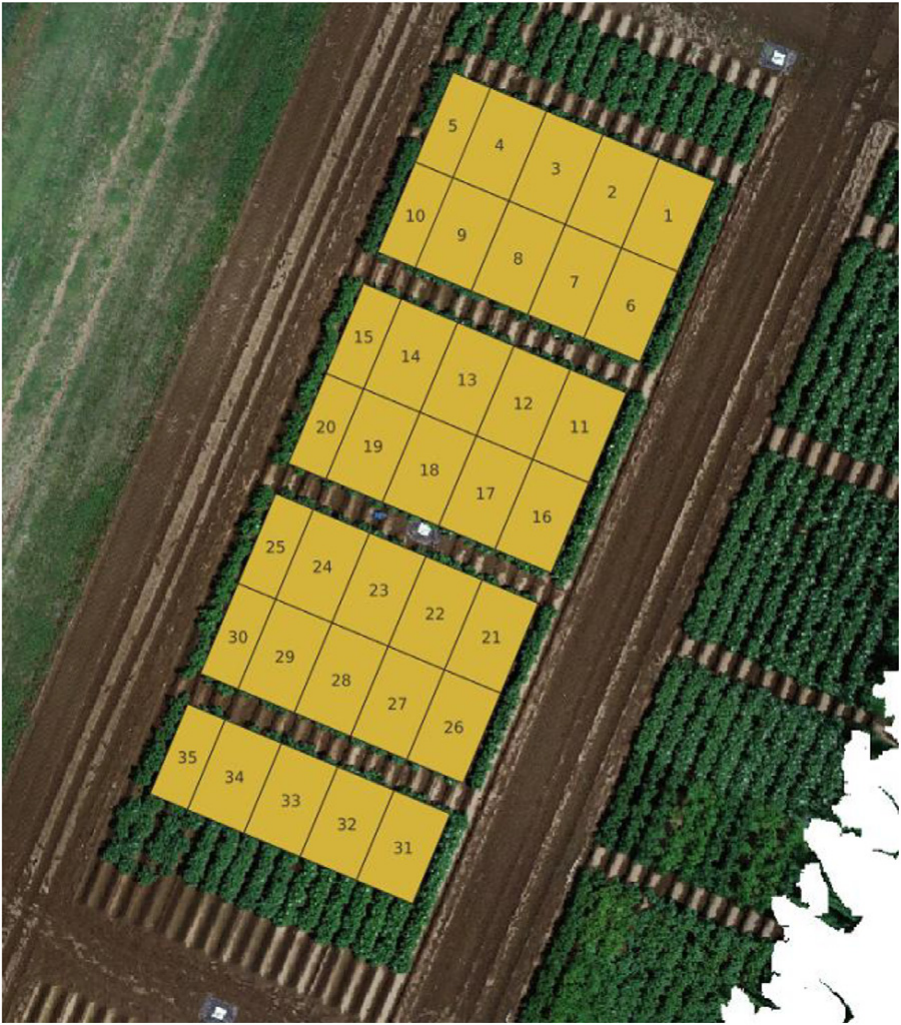
Field corresponding with studyId: TTADDA_NARO_2023.

The TTADDA_NARO_2023 study was conducted in the Memuro region of Japan (latitude: 42.888865279, longitude: 143.072670842). Most important properties are summarised in Table 3. Planting took place on May 18, 2023, and the harvest was completed on September 22, 2023. The field consisted of 35 plots (8 varieties), 5 plots with a size of 1.5 m x 3.6 m and 30 plots with a size of 2.25 m x 3.6 m. Plant density was 4.44 plants/m^2^. Similar to the previous years, the potatoes were hand planted with the sprout facing upwards at a spacing of 30 cm. After covering lightly with soil, a hiller was utilised to make a 30 cm high ridge along the planted potatoes and a ridge interval spacing of 75 cm. An overview of the field layout is shown in Fig. 5.

**Table 3:** Metadata of TTADDA_NARO_2023 field. *Values derived from SoilGrids [9].

Field properties	
studyId	TTADDA_NARO_2023
Number of plots	35
Number of varieties	8
Plant density [plants/m2]	4.44
Ground sampling distance [mm/pixel]	4.7
Number of drone flights	19
**Soil properties** [9]	
Clay [ %]	10.8*
Sand [ %]	55.6*
Silt [ %]	33.7*
pH [-]	5.8
Bulk density [kg/dm3]	0.74
Cation Exchange Capacity (CEC) [mmol+/kg]	186

### 4.3 TTADDA_WUR_2022

**Fig. 6:**
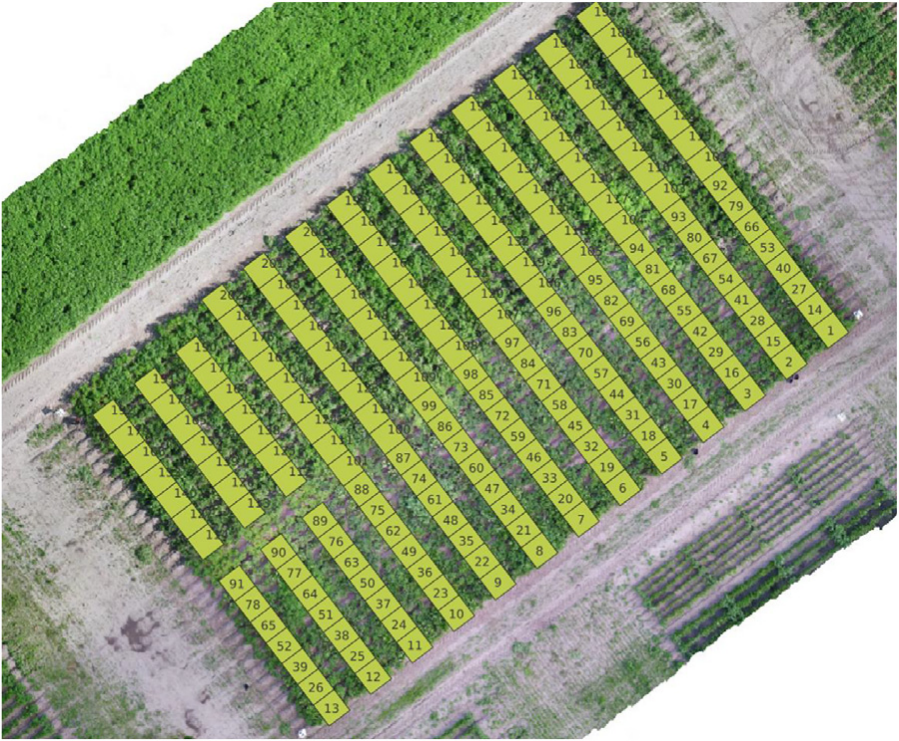
Field corresponding with studyId: TTADDA_WUR_2022.

The TTADDA_WUR_2022 study was conducted in the Wageningen the Netherlands (latitude: 51.99146594, longitude: 5.580833384). Most important properties are summarised in Table 4. Planting took place on April 19, 2022, and the harvest was completed on August 18, 2022. The field consisted of 202 plots (101 varieties), with a size of 1.5 m by 3 m including border plants. The net plot size that was used for phenotyping was 1.5 by 1.5 m Plant density was 5.33 plants/m^2^. The potatoes were hand planted with a planting distance of 0.25 m. After planting, ridges were formed over the tubers with a height of 0.30 m and 0.75 m distance between the ridges. An overview of the field layout is shown in Fig. 6.

**Table 4:** Metadata of TTADDA_WUR_2022 field.

Field properties	
studyId	TTADDA_WUR_2022
Number of plots	202
Number of varieties	101
Plant density [plants/m2]	5.33
Ground sampling distance [mm/pixel]	3.5
Number of drone flights	14
**Soil properties** [9]	
Clay [ %]	2
Sand [ %]	81
Silt [ %]	13
pH [-]	5.4
Bulk density [kg/dm3]	1.35
Cation Exchange Capacity (CEC) [mmol+/kg]	33

### 4.4 TTADDA_WUR_2023

**Fig. 7:**
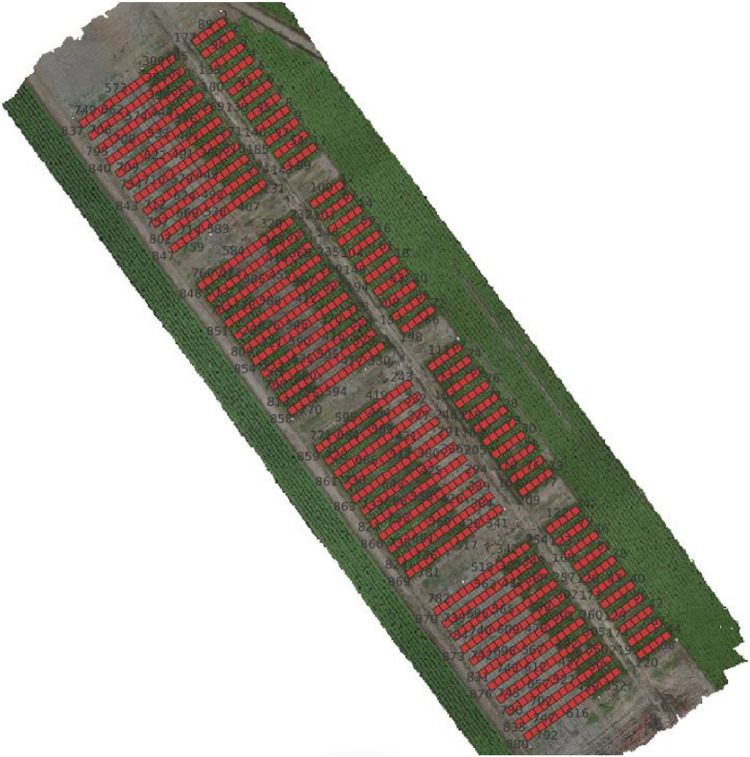
Field corresponding with studyId: TTADDA_WUR_2023.

The TTADDA_WUR_2023 study was conducted in the Wageningen the Netherlands (latitude: 51.9922293, longitude: 5.582514). Most important properties are summarised in Table 5. Planting took place on May 4, 2023, harvest was done 8 times biweekly, with the first harvest on May 31 and the eight harvest on September 6, 2023, in order to get intermediate data for each variety. The field consisted of 880 plots (55 varieties), with a size of 1.5 m by 3 m including border plants. The net plot size that was used for phenotyping was 1.5 m by 1.5 m. Plant density was 5.33 plants/m^2^. Plant density was 5.33 plants/m^2^. The potatoes were hand planted with a planting distance of 0.25 m. After planting, ridges were formed over the tubers with a height of 0.30 m and 0.75 m distance between the ridges. An overview of the field layout is shown in Fig. 7.

**Table 5:** Metadata of TTADDA_WUR_2023 field.

Field properties	
studyId	TTADDA_WUR_2023
Number of plots	880
Number of varieties	55
Plant density [plants/m2]	5.33
Ground sampling distance [mm/pixel]	2.0
Number of drone flights	16
**Soil properties**	
Clay [ %]	<1
Sand [ %]	85
Silt [ %]	11
pH [-]	6.1
Bulk density [kg/dm3]	1.35
Cation Exchange Capacity (CEC) [mmol+/kg]	67

## Limitations

The TTADDA-UAV dataset is comprehensive datasets, with five different studies, standardisation of measurements, collecting UAV data, creating orthomosaics, aligning ground truth data errors / mistakes can occur. By using the MIAPPE format all ground truth measurements were standardised, simplifying the dataset and making sure that all units are correct. Nevertheless, the perfect dataset does not exist. One important note is that the elevation maps of the TTADDA_WUR_2022 were not reliable. During the experiment orthomosaics were referenced using ground control points. Unfortunately, too few ground control points were used, as a result, depth data was inaccurate. The RGB data had sufficient data, therefore, the 2022 dataset was included.

## Ethics Statement

The authors declare that the ethical requirements of Data in Brief are considered. No human subjects, animals or data from any social media platform was included in this dataset.

## Data Availability

4TU.ResearchDataTTADDA-UAV: A Multi-Season RGB and Multispectral UAV Dataset of Potato Fields Collected in Japan and the Netherlands (Original data).
